# The burden attributable to headache disorders among children and adolescents in Mongolia: estimates from a national cross-sectional schools-based study

**DOI:** 10.1186/s10194-025-02202-0

**Published:** 2025-12-04

**Authors:** Otgonbayar Luvsannorov, Tsengunmaa Anisbayar, Munkhzul Davaasuren, Otgonzaya Baatar, Khaliunaa Batmagnai, Khulan Tumurbaatar, Sarantuya Enkhbaatar, Andreas Kattem Husøy, Derya Uluduz, Tayyar Şaşmaz, Elif Tuğçe Solmaz, Timothy J. Steiner

**Affiliations:** 1 Department of Neurology, Mongolian National University of Medical Sciences, Ulaanbaatar, Mongolia; 2 Department of Neuromedicine and Movement Science, Norwegian Centre for Headache Research (NorHead), Norwegian University of Science and Technology, Trondheim, Norway; 3 Department of Neurology and Clinical Neurophysiology, St Olavs University Hospital, Trondheim, Norway; 4 Neurology Department, Cerrahpaşa School of Medicine, Istanbul University, Istanbul, Turkey; 5 Public Health Department, School of Medicine, Mersin University, Mersin, Turkey; 6 Adana Provincial Health Directorate, Adana, Turkey; 7 Department of Neurology, University of Copenhagen, Copenhagen, Denmark; 8 Division of Brain Sciences, Imperial College London, London, UK

**Keywords:** Child and adolescent headache, Migraine, Tension-type headache, Medication-overuse headache, Undifferentiated headache, Burden of disease, Western Pacific Region, Mongolia, Global Campaign against Headache

## Abstract

**Background:**

Previously we have shown headache to be common among children (aged 6–11 years) and adolescents (aged 12–17) in Mongolia. This study estimates the headache-attributed burden from data collected in the same study. The purposes are to inform health and educational policies in Mongolia, and to enhance knowledge and understanding of the global burden of headache, particularly among these age groups.

**Methods:**

Following the generic protocol for *Lifting The Burden*’s global schools-based study, this cross-sectional survey administered structured questionnaires to pupils for mediated self-completion within classes in seven schools in four districts of the Capital city and three rural areas of Mongolia, selected to represent the country’s diversities. Headache diagnostic questions, except those for undifferentiated headache (UdH), were based on ICHD-3 criteria. Burden enquiry was conducted in multiple domains.

**Results:**

Among *N* = 4,266 school pupils, mean headache frequency was 4.1 days/4 weeks, of moderate average intensity and mostly short-lasting (mean 2.0 h). Mean proportion of time with headache was 1.2% (although 14.2% in those with probable medication-overuse headache [pMOH]). Symptomatic medication was taken for fewer than half of reported headaches. Pupils reported 2% of school time missed because of headache (17.5% in those with pMOH). The estimated risk of parental work loss in 4 weeks, due to a son’s or daughter’s headache, was 6.3% (15.6% for parents of children with migraine, 35.7% for pMOH). Emotional impact and quality-of-life scores both showed impact distinguishing between headache types (pMOH having greatest, followed by other headache on ≥ 15 days/month, migraine, tension-type headache and UdH).

**Conclusions:**

The headache-attributed burdens among children and adolescents may not, from this evidence, be deemed to be heavy (although losses from school time are not trivial). Nevertheless, there may be opportunity to avert at least some of the future need for health care among adults in Mongolia (one third of whom have headache with significant associated burden, mostly migraine or H15+, unquestionably calling for professional health care) by addressing the problem among children and adolescents. The short duration of headache among young people greatly limits the possibilities for acute intervention, shifting the emphasis in management onto lifestyle modification.

## Background

In an earlier publication we reported the prevalences of headache disorders among children (aged 6–11 years) and adolescents (aged 12–17 years) in Mongolia [1], a country in the Western Pacific Region (WPR) of the world. We had found the gender- and age-adjusted 1-year prevalence of any headache to be 59.4%, of migraine (definite + probable) 27.3%, of tension-type headache (TTH) (definite + probable) 16.1%, of undifferentiated headache (UdH) 6.6%, of all headache on ≥ 15 days/month (H15+) 4.2%, and of probable medication-overuse headache (pMOH) 0.7%. All headache types except UdH were more prevalent among females than males, and all were more prevalent among adolescents than children. Headache yesterday (HY) was reported by 15.9%, a proportion presumed to represent 1-day prevalence of any headache. In summary, we concluded, headache among these age groups in Mongolia was no less common than among adults [2].

UdH is a recently recognized disorder of young people essentially characterised by short duration (< 1 h) and believed to be expressions of migraine or TTH by the immature brain [3]. Other studies in Turkey [3], Lithuania [4], Austria [5], Ethiopia [6] and Zambia [7], all using the same methodology and questionnaire [8], have found UdH (defined in these studies as mild headache of duration < 1 h) to be more prevalent than migraine or TTH. We speculated that, in Mongolia, with its much lower prevalence of UdH than in these countries, there was reporting bias against relatively non-troublesome headache [1]. This explanation was supported by the relatively low estimate for all headache (59.4%), but it might also be that headache was more readily perceived to be of moderate intensity, therefore meeting criteria for probable migraine or probable TTH rather than UdH. In a study conducted subsequently in Nepal, where the same issue appeared to arise, we redefined UdH as *mild or moderate* headache of duration < 1 h [9], arguing that the distinction between mild and moderate pain, particularly in the minds of young people, was so subjective and arbitrarily made that it was unsatisfactory as a diagnostic criterion. This is an issue requiring resolution; here we retain the original definition of mild headache lasting less than an hour.

In this manuscript we report estimates of headache-attributed burden in these young people, who in latest estimates (in 2023) made up over one third of Mongolia’s population [10]. The data were collected at the same time, and from the same participants, as for the prevalence study [1]. These estimates are far more informative to health and educational policies than those of prevalence. While we analyse burden by diagnosis, the distinctions between UdH and probable migraine or probable TTH are not of great importance, given that headache disorders in children and adolescents are still evolving with brain maturation [3].

This is the first study from WPR within the global programme of similar studies supported by *Lifting The Burden* (LTB) as part of the Global Campaign against Headache. While it informs health and educational policies in Mongolia, its purposes include enhancement of knowledge and understanding of the global burden of headache, particularly among these age groups.

## Methods

The methods have been fully described previously [1], and are only summarised here. The study was a cross-sectional survey in schools selected to be nationally representative, following the generic protocol for LTB’s global programme [8]. Structured questionnaires were completed in hard copy by pupils in class, under supervision by their teachers and the investigators.

### Ethics and approvals

Approval was obtained from the research ethics committee of Mongolian National University of Medical Sciences (MNUMS).

School managers and teachers at selected schools agreed to participate, and prior verbal consent was obtained from each participating child or adolescent in accordance with the terms of ethics approval.

Data were collected anonymously. Data protection legislation was complied with.

### Sampling

Data collection was during a single academic term in 2018 in schools in seven districts of the country (four urban in the Capital city, Ulaanbaatar, and three rural [Khuvsgul, Tuv and Umnugobi aimaks]). We aimed thereby to capture the country’s socioeconomic diversity, and achieve representativeness with regard to types of school (government, private and mission schools) [1].

### Enquiry

We used the child and adolescent versions of LTB’s Headache-Attributed Restriction, Disability, Social Handicap and Impaired Participation (HARDSHIP) questionnaire [8] translated into Mongolian language. Demographic enquiry was followed by headache screening and diagnostic questions based, other than for UdH, on ICHD-3 criteria [11]. Burden enquiry in those reporting headache in the preceding year included symptom burden (frequency of headache, and its usual duration and intensity), symptomatic medication intake (frequency), headache-attributed lost time from schooling and other activities as well as lost parental work time, and selected (headache-relevant) questions from KINDL^®^ [12] addressing concentration, emotional impact and quality of life (QoL) [8]. QoL enquiry was addressed to all participants, with or without headache. Time frames of burden enquiry were the preceding 4 weeks, 1 week and 1 day (HY).

We transferred data into SPSS using independent double data-entry, reconciling discrepancies by reviewing source data.

### Analysis

We made diagnoses during analysis from responses to the diagnostic questions, following the HARDSHIP algorithm [8]. Among those reporting H15+, pMOH was diagnosed in those also reporting acute medication on ≥ 14 days in the preceding 4 weeks (assumed to equate to ≥ 15 days/month); otherwise, they were labelled “other H15+”. UdH was diagnosed before definite migraine, definite TTH, probable migraine and probable TTH. Definite and probable migraine were combined in subsequent analyses, as were definite and probable TTH, and are not separately reported here.

Headache frequency was reported as days in the preceding 4 weeks, medication intake as days in the preceding one week and 4 weeks. Headache intensity was reported on a scale of 1–3 (equating to response options “mild”, “moderate” and “severe”). Duration of episodes was reported in hours as < 1, 1–2, 2–4 or >4, converted for analysis by taking the mid-points (0.5, 1.5, 3 and 8 respectively, assuming a range of 4–12 h for the last). Proportion of time in ictal state (pTIS) was calculated, as a percentage, from frequency and duration. Lost health at individual level, also as a percentage, was calculated as pTIS*DW, where DW was the disability weight attributed to the ictal state of the headache type (migraine, TTH or pMOH) in the Global Burden of Disease (GBD) study 2013 (0–1, with 0 indicating no health loss and 1 indicating a health loss equivalent to being dead) [13]. Lost health at population level was estimated by factoring in prevalence.

We estimated lost school time in days in the preceding 4 weeks, counting not going to school as a whole day lost and leaving school early as a half-day lost, and calculated proportions using a denominator of 20. We separately counted days of limited activity (defined as “could not do things you wanted to because of your headaches”), with, for proportions, a denominator of 28. We counted parents of participating pupils who, according to the pupils, had lost time from their own work (regardless of quantity) during the same period. We calculated proportions reporting a lost school day because of HY, and predicted values of lost school yesterday from the product of number affected by headache and mean reported days (divided by 20) lost per pupil over the preceding 4 weeks. Responses to questions addressing concentration, emotional impact and QoL were all on a 4-point Likert scale (“never”, “sometimes”, “often”, “always”) [8], also referring to the preceding 4 weeks. We scored these 0–3, and summed them to generate an emotional impact score (including concentration; potential range 0–18, high being adverse) and a QoL score (0–36, low being adverse) [8].

We expressed proportions as %, with 95% confidence intervals (CIs), and used chi-squared tests for comparisons. We treated all other variables as continuous, and used descriptive statistics (means and standard deviations [SDs]). For comparisons of continuous data, we used ANOVA when these were distributed normally, and otherwise Kruskal-Wallis with post-hoc Dunn’s test. We considered *p* < 0.05 to be significant.

**Table 1:** Symptom burden by headache type and overall (*N* = 4,266)

Headache type	Pupils affected *n*	1-year prevalence^1^ %	Headache frequency days/4 weeks (mean±SD)	Headache duration hours (mean±SD)	Estimated proportion of time in ictal state %	Headache intensity scale 1–3^2^ (mean±SD)
**Episodic headaches**						
Migraine	1,195	27.3	3.5 ± 3.2	2.6 ± 2.5	1.4	2.2 ± 0.5
Tension-type headache	719	16.1	2.9 ± 3.0	1.9 ± 1.8	0.8	2.0 ± 0.4
Undifferentiated headache	290	6.6	1.7 ± 2.2	0.5 ± < 0.1	0.1	1.0 ± < 0.1
**Headache on ≥ 15 days/month**						
Probable medication-overuse headache	30	0.7	20.3 ± 4.3	4.7 ± 3.2	14.2	2.4 ± 0.5
Other headache on ≥ 15 days/month	157	3.5	18.5 ± 4.3	3.0 ± 2.8	8.3	2.2 ± 0.5
**All headache** ^**3**^	2,617	59.4	4.1 ± 5.1	2.0 ± 2.2	1.2	2.0 ± 0.6

Means are presented ± SDs; ^1^gender- and age-adjusted (data from [1]); ^2^equating 1–3 to the reported categories “mild”, “moderate” and “severe”, and treating as continuous data; ^3^includes unclassified headache

## Results

There were 4,266 participants (children 2,241 [52.5%], adolescents 2,025 [47.5%]) from 4,515 pupils present and eligible on survey days (participating proportion 94.5%). Of these, 2,107 (49.4%) were male, 2,159 (50.6%) female. Overall mean age was 11.3 ± 3.3 years (range: 6–17; median 11).

One-year prevalence of headache, adjusted for gender and age, was 59.4%, of migraine 27.3%, of TTH 16.1%, of UdH 6.6%, of pMOH 0.7% and of other H15 + 4.2%. There were 70 cases (1.6%) remaining unclassified. These findings, previously published [1], are repeated here because they are used in estimates of population burden.

### Symptom burden

Symptom burden was quantitatively assessed in terms of headache frequency, usual intensity and duration, and pTIS. All these are summarised, overall and by headache type, in Table 1, which also shows the previously published estimates of prevalence [1].

Mean headache frequency among those with any headache was 4.1 days/4 weeks. Migraine (3.5 days/4 weeks) was more frequent than TTH (2.9) or UdH (1.7; both *p* < 0.001), while pMOH (20.3 days/4 weeks) and other H15+ (18.5 days/4 weeks) were, inevitably, very much more frequent than the episodic headaches (*p* < 0.001) (Table 1). All episodic headaches were short-lasting: means were 2.6 h for migraine, 1.9 h for TTH and 0.5 h for UdH, these differing significantly from each other (*p* ≤ 0.001). pMOH (4.7 h) was somewhat longer lasting (*p* ≤ 0.001). Mean pTIS was 1.2% overall, 1.4% for migraine, less for TTH (0.8%) and much less for UdH (0.1%). Because both frequency and duration were higher, pTIS was much greater for pMOH (14.2%) and other H15+ (8.3%) (Table 1).

Mean headache intensity was 2.0 overall (moderate), with pMOH rated worst (2.4: moderate-to-severe; *p* < 0.001 *versus* TTH and UdH). Intensity was greater for migraine (2.2) than for TTH (2.0; *p* < 0.001). UdH, mild by definition, was rated less intense than all other headache types (all *p* < 0.001) (Table 1).

### Medication use

Table 2 shows reported use of symptomatic medication during the preceding week and 4 weeks, with headache frequencies during the preceding 4 weeks imported from Table 1 for ease of comparison. Responses to the two enquiries were reasonably consistent, albeit lower per week (except for pMOH) when dependent on recall over 4 weeks. Comparison of medication days with headache days over the preceding 4 weeks showed that (again except for pMOH) fewer than half of headaches were medicated. Other H15 + was notably untreated (Table 2) despite that, reportedly, its mean intensity was 2.2 and duration 3.0 h (Table 1).

**Table 2:** Symptomatic medication use by headache type and overall (*N* = 4,265^1^)

Headache type	Pupils responding *n*	Medication use days (mean ± SD)		Headache frequency days/4 weeks (from Table 1)
		Preceding week	Preceding 4 weeks	
**Episodic headaches**				
Migraine^1^	1,194	0.7 ± 1.1	1.7 ± 2.4	3.5 ± 3.2
Tension-type headache	719	0.4 ± 0.8	1.0 ± 1.9	2.9 ± 3.0
Undifferentiated headache	290	0.2 ± 0.6	0.5 ± 1.2	1.7 ± 2.2
**Headache on ≥ 15 days/month**				
Probable medication-overuse headache	30	4.0 ± 2.1	19.0 ± 3.6	20.3 ± 4.3
Other headache on ≥ 15 days/month	157	0.8 ± 1.4	2.4 ± 3.4	18.5 ± 4.3
**All headache** ^**1,2**^	2,616	0.6 ± 1.1	1.5 ± 2.9	4.1 ± 5.1

^1^One pupil did not respond to this enquiry; ^2^includes unclassified headache

### Headache-attributed lost health

Table 3 shows estimates of lost health at individual and population levels. Only pMOH was associated with an individual health loss of > 1%, but only migraine was responsible for > 0.1% of lost health distributed across the entire population of children and adolescents.

### Headache-attributed lost time

Table 4 shows pupils’ headache-attributed lost time from school and other activity, by age group, along with headache frequencies for comparison. Lost school time was from a total of 20 school days in 4 weeks, limited activity from an assumed total of 28 days.

Overall, pupils lost 0.4 days because of any headache, on average 0.1 days/week (2% of school time). Children lost twice as much time (0.6 days) as adolescents (0.3 days) (Table 4). A similar age differential applied to all headache types except pMOH. This was an outlier among headache types, accounting for 3.5 days lost (17.5% of school time), 5.6 (28.0%) among children and 1.7 (8.5%) among adolescents (Table 4).

**Table 3:** Headache-attributed lost health by headache type

Headache type	Disability weight (from [13])	Observed prevalence % (from [1])	Proportion of time in ictal state %		Lost health %	
			At individual-level^1^ mean (from Table 1)	At population level^2^	At individual level^1,3^ mean	At population level^4^
Migraine	0.441	28.0	1.4	0.4	0.6	0.17
Tension-type headache	0.037	16.9	0.8	0.1	0.03	0.005
Probable medication-overuse headache	0.223	0.7	14.2	0.1	3.2	0.02

^1^Among those reporting any headache in the preceding year; ^2^among all, with or without headache, calculated as the product of mean individual proportion of time in ictal state (pTIS) and prevalence; ^3^calculated as the product of mean individual pTIS and disability weight; ^4^among all, with or without headache, calculated as the product of mean individual lost health and prevalence

More days of limited activity were reported than were lost from school time (it should be noted that the questions were not phrased in order to exclude the latter from the former [8]). Overall, pupils lost a mean total of 1.6 days because of any headache, on average 0.4 days/week (5.7% assuming 28 days/4 weeks). Both pMOH and other H15+ stood out (Table 4). Children reported limited activity on more than half of days (15.3 days; 54.6%), three times as many as adolescents (5.1 days; 18.2%) (Table 4). Otherwise, adolescents generally reported more days of limited activity than children, albeit with small margins (1.7 *versus* 1.4 for all headache) (Table 4).

**Table 4:** Lost time from school and other activity because of headache in the preceding 4 weeks, by headache type and overall, and by age group (*N* = 4,266)

Headache type	Headache frequency days/4 weeks (mean)	Days of lost school	Days of limited activity
		days/4 weeks/affected pupil (mean ± SD)	
**Episodic headaches**			
Migraine children adolescents	3.5 3.1 3.8	0.5 ± 1.3 0.7 ± 1.5 0.4 ± 1.1	1.6 ± 2.1 1.4 ± 2.0 1.7 ± 2.2
Tension-type headache children adolescents	2.9 2.2 3.2	0.3 ± 0.8 0.3 ± 1.0 0.2 ± 0.7	1.0 ± 1.8 0.8 ± 1.6 1.2 ± 1.8
Undifferentiated headache children adolescents	1.7 1.4 1.8	0.2 ± 0.6 0.2 ± 0.7 0.1 ± 0.5	0.5 ± 1.1 0.4 ± 0.9 0.6 ± 1.2
**Headache on ≥ 15 days/month**			
Probable medication-overuse headache	20.3	3.5 ± 6.5	9.9 ± 10.2
children	22.1	5.6 ± 8.6	15.3 ± 11.0
adolescents	18.6	1.7 ± 2.9	5.1 ± 6.6
Other headache on ≥ 15 days/month	18.5	0.9 ± 2.8	5.1 ± 6.4
children	19.4	1.4 ± 3.1	5.3 ± 6.3
adolescents	18.2	0.7 ± 2.7	5.0 ± 6.4
**All headache** ^**1**^			
all children adolescents	4.1 3.5 4.5	0.4 ± 1.5 0.6 ± 1.8 0.3 ± 1.2	1.6 ± 2.9 1.4 ± 3.1 1.7 ± 2.8

^1^Includes unclassified headache

Although not all in the same direction, and with the exception of pMOH *versus* other H15+, all differences in days of limited activity between the headache types were significant (*p* < 0.01 for TTH *versus* UdH, otherwise *p* < 0.001 [Kruskal Wallis test]).

Lost school time yesterday because of HY, overall and by headache type, is shown in Table 5, with the not wholly reliable assumption that HY was the diagnosed headache type (no statistical comparisons are therefore made). Two further factors should be noted: the enquiry referred only to days of complete absence, and a school day could be lost yesterday only when yesterday happened to be a school day. The actual probability of missing a school day because of HY (among those reporting HY) was 3.8% overall, driven by migraine and other H15+ (numbers were small for UdH and pMOH).

Table 5 includes predictions based on recalled lost days/4 weeks (Table 4, column 3). The calculation for all headache, for example, was (0.4/20)*2,614 = 52 (7.7% of those reporting HY). All predicted values were higher than actual values (two-fold overall).

**Table 5:** Lost school day yesterday because of headache yesterday, by headache type and overall, with predictions based on recalled lost days/4 weeks

Diagnosed headache type	Pupils responding *n* ^1^	Proportion reporting HY (from [1]) *n* (% of those with headache type)	Missed school day yesterday *n* (% of those reporting HY)	
			Actual	Predicted
**Episodic headaches**				
Migraine	1,192	320 (26.8)	14 (4.4)	30 (9.4)
Tension-type headache	719	145 (20.2)	3 (2.1)	11 (7.6)
Undifferentiated headache	290	39 (13.4)	2 (5.1)	3 (7.7)
**Headache on ≥ 15 days/month**				
Probable medication-overuse headache	30	21 (70.0)	0 (0)	5 (23.8)
Other headache on ≥ 15 days/month	157	111 (70.7)	6 (5.4)	7 (6.3)
**All headache** ^**2**^	2,614	679 (26.0)	26 (3.8)	52 (7.7)

HY: headache yesterday; ^1^values reflect that not all pupils responded to every enquiry; ^2^includes unclassified headache

Table 6 shows numbers of parents who lost work at least once in the preceding 4 weeks because of their child’s headache. This table also includes headache frequencies for comparison. The risk of parental work loss was 6.3% overall, varying widely with headache type: 9.0% for migraine, a little over 3% for TTH and UdH, but 23.3% for pMOH. The essential finding was that risk was strongly and negatively age-related (for all headache, chi-squared = 82.69; *p* < 0.001), reflected in all headache types (Table 6).

**Table 6:** Parents’ lost work in the preceding 4 weeks, by pupils’ headache type and overall, and by age group

Headache type	Pupils responding *n*	Headache frequency days/4 weeks	Parent missed work *n* (% of pupils with headache type)
**Episodic headaches**			
Migraine	1,193	3.5	109 (9.1)
children	494	3.1	77 (15.6)
adolescents	699	3.8	32 (4.6)
Tension-type headache	719	2.9	24 (3.3)
children	226	2.2	18 (8.0)
adolescents	493	3.2	6 (1.2)
Undifferentiated headache	290	1.7	10 (3.4)
children	127	1.4	7 (5.5)
adolescents	163	1.8	3 (1.8)
**Headache on ≥ 15 days/month**			
Probable medication-overuse headache	30	20.3	7 (23.3)
children	14	22.1	5 (35.7)
adolescents	16	18.6	2 (12.5)
Other headache on ≥ 15 days/month	157	18.5	11 (7.0)
children	38	19.4	6 (15.8)
adolescents	119	18.2	5 (4.2)
**All headache** ^**1**^ children adolescents	2,614 981 1,633	4.1 3.5 4.5	165 (6.3) 116 (11.8) 49 (3.0)

^1^Includes unclassified headache

### Emotional impact and quality of life (QoL)

Scores were not normally distributed on either test. Nevertheless, we calculated means and SDs for each headache type, and for no headache on the QoL score (Table 7). Neither score had absolute meaning, but both distinguished between (most) headache types and, for QoL, no headache.

Thus, while pMOH and other H15+ were not significantly different from each other, both (with scores of 10.1 and 8.3/18 respectively) had greater emotional impact (*p* < 0.001) than all other headache types. Of the episodic headaches, migraine had greatest impact (7.0), followed by TTH (5.9), with UdH least (4.3; all *p* < 0.001) (Table 7).

Despite a score of only 24.8/36 for those without headache, the QoL scale was quite sensitive to headache, with only UdH (24.2/36) having insignificant impact (Table 7). pMOH had greatest impact (18.2), followed by other H15+ (20.0), these two again not significantly different from each other. Migraine (22.0) had greater impact than TTH (23.4) and UdH (both *p* < 0.001) (Table 7).

**Table 7:** Emotional impact and quality of life scores by headache type

Headache type	*n* ^1^	Score (mean ± SD)	Between-type significance (Kruskal-Wallis with post-hoc Dunn’s test)			
			TTH	UdH	pMOH	Other H15+
**Emotional impact** (0–18; higher scores adverse)						
Migraine	1,195	7.0 ± 2.9	*p* < 0.001	*p* < 0.001	*p* < 0.001	*p* < 0.001
TTH	719	5.9 ± 2.7		*p* < 0.001	*p* < 0.001	*p* < 0.001
UdH	290	4.3 ± 2.5			*p* < 0.001	*p* < 0.001
pMOH	30	10.1 ± 2.6				*p* > 0.05
Other H15+	157	8.3 ± 2.8				
All headache^2^	2,617	6.4 ± 3.0				
**Quality of life** (0–36; lower scores adverse)						
No headache	1,644	24.8 ± 4.3	*p* < 0.001 against all headache types except UdH (*p* > 0.05)			
Migraine	1,189	22.0 ± 4.6	*p* < 0.001	*p* < 0.001	*p* < 0.05	*p* < 0.001
TTH	718	23.4 ± 4.2		*p* > 0.05	*p* < 0.001	*p* < 0.001
UdH	288	24.2 ± 4.3			*p* < 0.001	*p* < 0.001
pMOH	30	18.2 ± 5.3				*p* > 0.05
Other H15+	157	20.0 ± 4.7				

TTH: tension-type headache; UdH: undifferentiated headache; pMOH: probable medication-overuse headache; ^1^values reflect that not all pupils responded to every enquiry; ^2^includes unclassified headache

## Discussion

Our previously published study showed headache to be common among children and adolescents in Mongolia [1]. This manuscript adds data, collected in the same study, on the attributed burden. These data are more informative for public-health policy than prevalence estimates alone. They may also be relevant to educational policy.

In summary, among a representative sample of *N* = 4,266 school pupils, we found a mean headache frequency of 4.1 days/4 weeks, with a mean headache intensity of 2.0 (moderate). Most were short-lasting (mean 2.0 h), so that overall mean pTIS was only 1.2%, although much greater in those with pMOH (14.2%) or other H15+ (8.3%). Symptomatic medication was taken for fewer than half of reported headaches (on average less than once per week). Nevertheless, pupils with headache reported an overall loss of 2% of their school time (children twice as much as adolescents), those with pMOH 17.5% (children more than three times as much as adolescents). For those with HY (*n* = 679 [26.0% of those with headache in the last year]), the actual probability of missing a school day was 3.8% overall, less than predicted from recall over 4 weeks. This suggests losses were overestimated on recall, although caution is demanded when interpreting data from a single day. There were more days of limited activity (including play time) than were reportedly lost from school time, with slight reversal of the age difference – the speculative explanation being that school time assumes greater relative importance among adolescents.

An often-hidden burden of headache in young people is the impact on their parents. Here we estimated an overall risk of parental work loss in 4 weeks of 6.3%. More than one in seven parents of children with migraine (15.6%) lost some time from their own work, and more than one in three (35.7%) parents of children with pMOH. While both prevalence and all other measures of burden were higher among adolescents than among children, parental work loss declined, by about three quarters in relative terms, almost certainly a reflection of diminishing dependence with age.

Telling evidence that the burden of headache is not trivial among children and adolescents is in the emotional impact and QoL scores. Neither had absolute meaning, but both showed impact distinguishing between headache types, with pMOH having greatest, followed, much as expected, by other H15+, migraine, TTH and UdH. Only UdH had no measurable impact on QoL (not significantly lower than in those with no headache).

There are no similar data for these age groups from the enveloping (but ethnically and culturally distinct) countries of Russia and China, or from any other country in WPR, but we do have data from an adult population-based study (*N* = 2,043) conducted in Mongolia in 2017 [2]. Age- and gender-adjusted prevalences were 23.1% for migraine, 29.1% for TTH, 5.7% for pMOH and 5.0% for other H15+ [2]. HY was reported by 20.1%. Those reporting any headache in the last year (*n* = 1,351) (spending, on average, 9.7% of all their time with headache), lost 1.3 days/3 months from income-generating work and 2.4 days/3 months from household work (essential chores of daily living). Our estimates in this study were per 28 days; if multiplied by 13/4, they become 1.3 days/3 months lost from school (exactly matching adults’ lost days from paid work) and 5.2 days of limited activity (which is not the same as lost days). The very big difference between adults and the young people studied here is in pMOH, with its high associated individual burden: prevalence estimates of 5.7% [2] and 0.7% respectively. The latter increased somewhat with age, from 0.6% to 0.8%, in our earlier findings [1]. What is concerning, and attracted our attention previously, is the increasing prevalence of other H15+, from 1.7% among children to 5.9% among adolescents [1]. The genesis of adult levels of pMOH may well lie here.

Our needs assessment among adults in Mongolia estimated that one third (33.2%) of the population have headache with high associated burden requiring professional health care (mostly migraine or H15+) [2]. From the evidence in the present study, the headache-attributed burdens among children and adolescents, while not trivial, are nowhere near as heavy. The estimated population-level health loss (using DWs from GBD [13]) was only 0.17% for migraine and far less for TTH (which has a very low DW) and pMOH (with a very low prevalence [0.7%]), although an important caveat here is that the DWs from GBD were developed only with adults in mind [13]. Nevertheless, there may be opportunity to avert at least some of the future adult need for health care by addressing the problem among children and adolescents.

One reason why headache is so often neglected among young people is its short duration (mean 2.0 h), also noted in other studies [3–7, 9]. It is this, in particular, that creates diagnostic uncertainty [1, 3]. It also greatly limits the possibilities for acute intervention, shifting the emphasis in management onto lifestyle modification.

The strengths of this study were in the use of standardised methodology, a large sample size (*N* = 4,266) derived from nationwide sampling, and a high participating proportion (94.5%). The limitations inherent in cross-sectional surveys were present. We assisted younger children who had difficulties in understanding or answering questions through mediated enquiry (supervised completion of questionnaires in class) [1, 8]. We countered uncertainties in recall over the past 4 weeks by separate enquiry into HY. There may have been an underlying limitation in that the ICHD criteria do not apply well to children and adolescents [14], which we believe underpins the importance of including UdH as a separate diagnosis. However, the definition of UdH may need revision [9], which would affect our prevalence estimates and, accordingly, our burden estimates for each diagnosis. As noted, with regard to burden attribution, the distinctions between UdH and probable migraine or probable TTH may not be of great importance, since headache disorders in children and adolescents are still evolving with brain maturation [3]. Diagnosis by questionnaire of H15+, beyond association with medication overuse (pMOH), remains problematic and unreliable: even in clinic, this usually requires diary-assisted follow-up. Estimates of the burdens attributable to migraine and TTH did not, therefore, include chronic migraine or TTH. These, if present, would have been among the 4.2% with other H15+ [1].

## Conclusions

While the measurable headache-attributed burdens among children and adolescents in Mongolia may not be heavy, the emotional impact and QoL scores provide evidence that the overall burden of headache is not trivial. Losses from school time (2% overall, 17.5% among those with pMOH) are also not to be regarded as insignificant. Addressing headache among these age groups may avert at least some of the future high need for health care among adults [2]. These are messages for health and educational policies in Mongolia.

## Data Availability

The data are held on file at the University of Mersin and at Norwegian University of Science and Technology. Once analysis and publications are completed, they will be freely available for non-commercial purposes to any person requesting access in accordance with the general policy of the Global Campaign against Headache.

## Abbreviations

ANOVA: Analysis of variance
DW: Disability weight
GBD: Global Burden of Disease (study)
H15+: Headache on ≥ 15 days/month
HARDSHIP: Headache-Attributed Restriction, Disability, Social Handicap and Impaired Participation (questionnaire)
HY: Headache yesterday
ICHD: International Classification of Headache Disorders
MOH: Medication-overuse headache
pMOH: Probable MOH
MNUMS: Mongolian National University of Medical Sciences
pTIS: Proportion of time in ictal state
QoL: Quality of life
SD: Standard deviation
TTH: Tension-type headache
UdH: Undifferentiated headache
WPR: Western Pacific Region
